# Ascitic complement system in ovarian cancer

**DOI:** 10.1038/sj.bjc.6602334

**Published:** 2005-02-22

**Authors:** L Bjørge, J Hakulinen, O K Vintermyr, H Jarva, T S Jensen, O E Iversen, S Meri

**Affiliations:** 1 Department of Bacteriology and Immunology, Haartman Institute, University of Helsinki, FIN-00014 Helsinki, Finland; 2 Department of Obstetrics and Gynaecology, N-5021 Haukeland Hospital, University of Bergen, Norway; 3 Department of Microbiology and Immunology, The Gade Institute, Haukeland University Hospital, N-5021 Bergen, Norway; 4 Department of Pathology, The Gade Institute, Haukeland University Hospital, N-5021 Bergen, Norway; 5 Huslab, Helsinki University Central Hospital, FIN-00290 Helsinki, Finland

**Keywords:** ovarian neoplasm, ascitic fluid, complement, immunosurveillance, immunotherapy

## Abstract

Ovarian cancer spreads intraperitoneally and forms fluid, whereby the diagnosis and therapy often become delayed. As the complement (C) system may provide a cytotoxic effector arm for both immunological surveillance and mAb-therapy, we have characterised the C system in the intraperitoneal ascitic fluid (AF) from ovarian cancer patients. Most of the AF samples showed alternative and classical pathway haemolytic activity. The levels of C3 and C4 were similar to or in the lower normal range when compared to values in normal sera, respectively. However, elevated levels of C3a and soluble C5b-9 suggested C activation *in vivo*. Malignant cells isolated from the AF samples had surface deposits of C1q and C3 activation products, but not of C5b-9 (the membrane attack complex; MAC). Activation could have become initiated by anti-tumour cell antibodies that were detected in the AFs and/or by changes on tumour cell surfaces. The lack of MAC was probably due to the expression of C membrane regulators CD46, CD55 and CD59 on the tumour cells. Soluble forms of C1 inhibitor, CD59 and CD46, and the alternative pathway inhibitors factor H and FHL-1 were present in the AF at concentrations higher than in serum samples. Despite the presence of soluble C inhibitors it was possible to use AF as a C source in antibody-initiated killing of ovarian carcinoma cells. These results demonstrate that although the ovarian ascitic C system fails as an effective immunological surveillance mechanism, it could be utilised as an effector mechanism in therapy with intraperitoneally administrated mAbs, especially if the intrinsic C regulators are neutralised.

Ovarian cancer is the sixth most common malignant neoplasm in women worldwide (Pisani *et al*, 2002). Despite maximal cytoreductive surgery and chemotherapeutic regimens, the overall survival rate is still below 40% (Bjørge T *et al*, 1998). This is mainly due to innate or acquired drug resistance and the fact that the majority of tumours have progressed to an advanced stage at the time of diagnosis (Bjørge T *et al*, 1998; Baekelandt *et al*, 2000).

Ovarian carcinomas are the most common primary tumours that lead to the production of free abdominal fluid or ascites (AF) (Zeimet *et al*, 1998). However, the mechanisms of generation and the role of ascites in the biology of ovarian cancer are widely unknown. Usually, at the time of diagnosis, the cancer has already spread beyond the ovaries, and symptoms related to the presence of free abdominal fluid are often the first manifestation of the disease (Zeimet *et al*, 1998). Characteristically, the malignant cells often remain confined to the abdominal cavity in direct contact with AF both as peritoneal implants and as free-floating tumour cells, thereby complicating the complete surgical removal of the tumour cells (Zeimet *et al*, 1998).

The complement (C) system is a major component of innate immunity. It eliminates invading microorganisms, transformed cells and molecular aggregates from tissues and biological fluids (Walport, 2001). Complement activation occurs through three convergent pathways, in which the C components are usually activated by sequential proteolytic cleavages and/or by binding to previously activated components. This results in the release of chemotactic factors and cell-activating anaphylatoxins, deposition of opsonic fragments and formation of the membrane attack complex (MAC) (Walport, 2001). Although C may become activated on tumour cells by tumour-binding antibodies (Magyarlaki *et al*, 1996), immune complexes (Lucas *et al*, 1996) or as a consequence of apoptosis (Matsumoto *et al*, 1997) or proteolytic processes (Niculescu *et al*, 1992; Yamakawa *et al*, 1994; Bjørge L *et al*, 1997a), the cytotoxic activity of C is insufficient as an effective immunological surveillance mechanism against tumours (Gorter and Meri, 1999; Jurianz *et al*, 1999). Immunotherapy with C-activating mAbs in the treatment of solid tumours has so far shown only limited success (Dillman, 1994; Gruber *et al*, 1996). The ‘humanised’ anti-CD20 mAb, rituximab has shown an overall response rate of over 50% (Maloney *et al*, 1997) in the treatment of non-Hodgkin's lymphomas. This mAb acts by activating C and by causing antibody-dependent cellular cytotoxicity and apoptosis (Golay *et al*, 2000; Harjunpaa *et al*, 2000). The limited susceptibility of solid tumour cells to C, can, in part, be explained by the presence of intrinsic cellular C resistance mechanisms and by limited accessibility of mAbs and C components to the tumours (Bjørge L *et al*, 1997a; Hakulinen and Meri, 1998; Gorter and Meri, 1999; Jurianz *et al*, 1999).

Complement resistance of both malignant tumour cells and normal cells is mediated by membrane-bound C regulatory proteins that either limit the formation of the C3/C5 convertase enzymes, or the assembly of the MAC (Morgan and Meri, 1994; Gorter and Meri, 1999; Jurianz *et al*, 1999; Walport, 2001). The key C enzymes, C3/C5 convertases, are inhibited by the following C regulatory proteins; complement receptor type 1 (CR1, CD35), decay-accelerating factor (DAF, CD55) and membrane cofactor protein (MCP, CD46). These molecules prevent the assembly and promote the decay of the C3/C5 convertase complexes (CR1 and DAF), or serve as cofactors for the plasma serine protease factor I, which irreversibly inactivates C4b and C3b (CR1 and MCP). Protectin (CD59), on the other hand, inhibits the formation and function of MAC complexes on cell surfaces. In addition, nucleated cells have the capacity to remove MAC-containing membrane particles from their cell surfaces by vesiculation or internalisation (Morgan, 1992). Some tumour cells have also been found to secrete the soluble C regulators factor H (FH) and factor H-like protein 1 (FHL-1) (Junnikkala *et al*, 2000, 2002).

Antibody responses develop in ovarian cancer, and tumour-reactive immunoglobulins directed both against intracellular proteins and surface antigens have been demonstrated in patient sera (Chinni *et al*, 1997; Taylor and Gercel-Taylor, 1998; Abendstein *et al*, 2000; Gercel-Taylor *et al*, 2001). The antibody levels increase as the disease progresses (Taylor and Gercel-Taylor, 1998). Furthermore, ascitic immunoglobulins of IgM and IgG classes have been described (Silburn *et al*, 1984a, 1984b; Rao and Hanjani, 1988). They are present either as circulating free antibodies, in immune complexes, or bound to tumour cells and cellular fragments (Silburn *et al*, 1983; Runowicz *et al*, 1989; Kawata and Sekiya, 1998; Taylor and Gercel-Taylor, 1998). Despite the fact that they are partially tumour-reactive, they fail to destroy the tumour cells (Taylor and Gercel-Taylor, 1998).

Factors of the C system (Runyon *et al*, 1985; Mal *et al*, 1991; Alexandrakis *et al*, 2001), chemoattractants and opsonic activity (Runyon *et al*, 1985; Mal *et al*, 1991) have been described in AF from ovarian cancer patients. The ascitic C system, although incompletely characterised, has been given the role as a protector against spontaneous bacterial peritonitis (Runyon *et al*, 1985).

In our previous studies, we have described the expression of the C regulators MCP, DAF and CD59 on ovarian carcinoma cells both in solid tumours and in culture (Bjørge L *et al*, 1997a). DAF and CD59 appeared to be the main factors that protected malignant ovarian cells against C-mediated killing. Furthermore, FH and FHL-1 were found to be produced by ovarian tumour cells, and in solid ovarian tumours FH and FHL-1 were present in the apical tumour cell layers (Junnikkala *et al*, 2002).

It has been suggested that the AF represents an appropriate environment for studies of tumour biology and locally occurring host–tumour interactions (Zeimet *et al*, 1998). In the present study, we have investigated the AF C system in ovarian cancer patients to more precisely understand the local humoral antitumour immune responses, and to reveal mechanisms whereby the growing tumours escape the host's innate immune system. As intraperitoneal delivery of mAbs could potentially be used in the treatment of ovarian cancer (Rubin, 1993), the susceptibility of malignant ovarian cells to C-mediated killing with AF as a C source was also examined.

## MATERIALS AND METHODS

### Collection and storage of AF samples and preparation of ascitic tumour cells

The study was approved by the Regional Ethical Committee of Western Norway (Jnr. 234/97-81.97), and informed consents were obtained from the patients. Ascitic fluid samples were obtained in a prospective, nonselective fashion from patients referred to the Haukeland University Hospital (Bergen, Norway) for the treatment of presumed ovarian cancer. The histopathological diagnoses were obtained according to the 1986 revised staging system of the Fédération International d’Obstétrique (FIGO, 1987), at the Department of Pathology, The Gade Institute, Haukeland University Hospital. Ascitic fluid samples were obtained during laparotomy immediately after the peritoneal cavity was opened and taken into silicone-coated tubes or tubes containing 1 mM EDTA (final concentration). The samples were cooled to 4°C and transported to the laboratory. The samples from the patients (*n*=16) (Table 1) were centrifuged at 1100 *g* at 4°C for 10 min to separate cells and other debris from the fluid phase. The cell-free fluid was aliquoted and stored at −70°C until used. Samples from two patients with nonmalignant AF were obtained by paracentesis at the Medical Department, Division of Gastroenterology, Haukeland University Hospital, and processed in the same way as the AF from the patients with ovarian tumours.

Tumour cells were isolated from AF samples from two patients (patients no. 2 and 10) by a density gradient separation method. Conically shaped glass tubes (15 ml; Schott Glasswerke, Mainz, Germany) or plastic vials (50 ml; Elkay, Shrewsbury, USA) were used for the preparation of density gradients. The density separation liquid (Lymfoprep solution®; Nycomed, Oslo, Norway) was loaded in three equally sized layers. The bottom layer consisted of Lymfoprep solution® (1.077 g ml^−1^), the middle layer (1.059 g ml^−1^) of a mixture of 2.25 vol. Lymfoprep® and 0.75 vol. Krebs Hepes Ringer (KHR) solution (136.9 mM NaCl, 5.36 mM KCl, 0.34 mM Na_2_HPO_4_·2H_2_O, 0.35 mM KH_2_PO_4_, 0.8 mM MgSO_4_·7H_2_O, 1 mM Hepes, pH 7.4) without Ca^2+^, and the top layer (1.030 g ml^−1^) of a mixture of 1 vol. Lymfoprep® and 2 vol. of the KHR solution. All density gradient fractions were supplemented with 1 mM EDTA. The AF samples used for cell isolation were all supplemented with 1 mM EDTA and kept at room temperature all the time. The AF was carefully loaded onto the density gradients and centrifuged at 1500 *g*_av_ for 20 min. After centrifugation, several cell bands appeared. The main tumour cell fraction (T-fraction) was found at the interface between the upper and middle density fractions as a clear band in samples having many tumour cells. At the interface between the middle and lower gradients, most of the inflammatory cells (B-fraction) were found, along with a varying degree of tumour cells. Routinely, the T- and B-fractions were collected as they contained more than 80% of all tumour cells. Small aliquots of cells from the T- and B-fractions were routinely spun on a cytocentrifuge (1250 r.p.m., 10 min), and the cells were mounted on silicone-coated microscope slides (Dako, Glostrup, Denmark). The samples were fixed in acetone (Merck, Darmstadt, Germany) for 20 min and air-dried. The cells were identified as tumour cells by cytology (staining with Diff Quick and Giemsa), and by immunocytochemistry staining for the tumour antigens CA125 and Ber-EP4.

### Antibodies and sera

A rat hybridoma cell line (YTH53.1) producing a mAb to human CD59 was originally obtained from Professor H Waldmann (Sir William Dunn School of Pathology, Oxford, UK). YTH53.1 (IgG2b) was purified from cell culture supernatant using a Protein G Sepharose 4 Fast Flow affinity column (Pharmacia BioTech, Uppsala, Sweden). Mouse mAbs to DAF (CD55; BRIC230 and BRIC216; IgG1) and CD59 (BRIC229; IgG2b) were purchased from the International Blood Group Reference Laboratory (IBGRL, Bristol, UK). Murine mAb to human MCP (CD46; J4-48; IgG1) was purchased from Immunotech (Marseille, France) or obtained as a gift (GB24; IgG1) from Drs K Liszewski and JP Atkinson (Washington University School of Medicine, St Louis, MO, USA). Murine mAb to human TCC (aE11; IgG2a) and rabbit polyclonal antibodies to human C1q, C3c, IgG and IgM were all obtained from Dako. Peroxidase-conjugated rabbit immunoglobulins (Igs) to mouse IgG, peroxidase-conjugated-swine Igs to rabbit IgG, peroxidase-conjugated rabbit Igs to human IgG and peroxidase-conjugated rabbit Igs to human IgM were purchased from Jackson ImmunoResearch Laboratories (Burlingame, CA, USA). Normal human serum (NHS) that served as a source of C was prepared from coagulated blood of healthy laboratory personnel and stored in aliquots at −70°C until used.

### Cell lines

The human ovarian adenocarcinoma cell lines (Caov-3, SK-OV-3 and SW626), human ovarian teratocarcinoma cell line (PA-1) and human endothelial cell line (HUV-EC-C) were all obtained from American Type Culture Collection (ATCC, Manassas, VA, USA). The cells were maintained in Dulbecco's modified Eagle's medium (DMEM; Gibco, Paisley, UK) supplemented with 10% heat-inactivated foetal calf serum (FCS; Gibco), 2 mM L-glutamine (Bio Whittaker, Walkersville, MD, USA) and antibiotics (10 U ml^−1^ of penicillin (AL, Oslo, Norway) and 10 *μ*g ml^−1^ of streptomycin (Glaxo, Greenford, UK)) at 37°C in a humidified atmosphere with 5% CO_2_. Cells were grown in 75 cm^2^ cell culture flasks (Costar, Cambridge, MA, USA) and subcultured twice a week. Suspensions of the cells were obtained by incubating the cell cultures with Versene/EDTA (Gibco). Thereafter, the cells were washed twice in growth medium and resuspended.

Secreted or shed molecules from the different cell lines were collected in the following way: the cell lines were grown in 75 cm^2^ cell culture flasks until 90% confluent, washed three times with a cell culture medium containing no FCS, and fresh medium without supplements was added. After a 48-h incubation period, the cell cultures were harvested and centrifuged twice at 500 and 800 *g* for 10 min, before the supernatants were filtered through ultrafilters with a pore size of 0.45 *μ*m (Minisart®, Sartorius AG, Göttingen, Germany). The samples were further concentrated (100 ×) in Biomax concentrators (Millipore, Bedford, MA, USA) with a cutoff limit of 10 kDa.

### Concentrations of IgM, IgG, C3, C4, C1-inhibitor and factor H in AF

The concentrations of IgM, IgG, C3 and C4 were determined using precipitating antisera in a Nephelometer Analyzer (Behringwerke AG, Marburg, Germany). The C1-inhibitor (C1INH) concentration was determined by radial immunodiffusion (Nor-Partigen; Behringwerke AG). Factor H and FHL-1 concentrations in the AF samples were determined by an enzyme-linked immunoabsorbent assay (ELISA) as described previously (Junnikkala *et al*, 2002). Samples of AF were assayed for total protein content using the Bio-Rad Protein Assay (Bio-Rad Laboratories, Hercules, CA, USA) according to the instructions from the manufacturer.

### CD59, DAF and MCP in AF samples and in cell culture supernatants

Ascitic fluid samples and concentrated cell culture supernatants were electrophoresed in 10% SDS–PAGE slab gels under nonreducing conditions. The proteins were transferred onto nitrocellulose filters. After blocking the membranes with 5% nonfat milk powder in phosphate-buffered saline (PBS), pH 7.4 for 30 min, the filters were incubated overnight at +4°C with mAbs directed against CD46 (J4-48; 0.5 *μ*g ml^−1^), CD55 (BRIC230 or BRIC216; 0.5 *μ*g ml^−1^) or CD59 (BRIC229; 0.5 *μ*g ml^−1^) diluted in PBS containing 5% nonfat milk power. In controls, the primary antibodies were omitted or replaced with an irrelevant antibody of the same subclass as the specific mAb. After washing with PBS, the bound antibodies were visualized using peroxidase-conjugated anti-mouse IgG and a chemiluminescence substrate (SuperSignal® ULTRA Substrate; Pierce Chemical Company, Rockford, IL, USA) according to the instructions from the manufacturer.

### Analysis of relative amounts of soluble and membrane forms of CD59 in AF

Triton X-114 (TX-114) phase separation was used to separate the soluble and phospholipid-anchored forms of CD59 in AF (Lehto and Meri, 1993). TX-114 (Sigma Chemical Co, St Louis, MO, USA) was added at a concentration of 0.5% to cell-free AF at +4°C from 10 patients (seven patients with ovarian cancer and three patients with other gynaecological diseases). The samples were incubated at 37°C for 3 min until clouding occurred. The detergent and aqueous phases were separated by centrifugation at 3000 *g* for 3 min at +22°C. Samples of the different phases were analysed by SDS–PAGE and immunoblotting for the presence of CD59 as described above. The aqueous phase contained exclusive CD59 that was fee of attached phospholipids.

### Complement activation *in vivo* and haemolytic C activity in AF

*In vivo* C activation in AF samples containing 1 mM EDTA was determined by analysing levels of C3a and TCC (SC5b-9). C3a levels were measured using an ELISA- kit from Quidel according to the instructions from the manufacturer. SC5b-9 concentrations were determined by an ELISA assay as described previously (Lehto and Meri, 1993).

The haemolytic activities of the alternative and classical C pathways in AF samples (without additives) were determined by radial diffusion in gels containing antibody-coated chicken or guinea pig erythrocytes, respectively (The Binding Site Ltd., Birmingham, England). Serial dilutions of an NHS pool with known C activity were used to generate standard curves.

### Ascitic fluid C-mediated lysis of ovarian tumour cells

Single cell suspensions of the PA-1 and Caov-3 cell lines were labelled with sodium-chromate (^51^Cr) by incubating 6 × 10^6^ cells with 40 *μ*Cu of ^51^Cr (Amersham, Cardiff, UK) in 1 ml of DMEM for 2 h at +37°C. After incubation, the cells were washed three times in DMEM. To remove unbound ^51^Cr, the cells were further incubated in 1 ml of DMEM for 30 min at +37°C. After two washes in DMEM, the cell number was adjusted to 2 × 10^6^ cells ml^−1^. Duplicate aliquots (50 *μ*l) of the ^51^Cr-labelled cells were incubated for 15 min at room temperature with appropriate dilutions of the YTH53.1 mAb in 100 *μ*l. Aliquots of AF (from different patients) or NHS at different dilutions were added, and the cells were further incubated for 60 min at +37°C. The radioactivity released into the medium was counted from 100 *μ*l portions of the supernatants. Final concentrations of the reagents are provided in the respective figure legends. In controls, DMEM was used instead of the reactants. The maximum ^51^Cr release was obtained from cells exposed to 1.0% Nonidet-P40 (NP-40). Spontaneous ^51^Cr release was counted from the supernatants of the cells incubated with the cell culture medium only. The cell lysis was calculated as a percentage of the maximum released radioactivity: ((released radioactivity−spontaneous release)/(maximum released radioactivity−spontaneous release)) × 100%.

### Immunohistochemistry analysis

Cytospin slides made from AF samples from patients no. 2 and 10 were initially immersed in PBS containing 1% BSA for 5 min to rehydrate the sections. They were further incubated with 0.3% H_2_O_2_ diluted in PBS for 15 min. The cytospin slides were then incubated with 10% heat-inactivated NHS diluted in PBS for 30 min at room temperature, before being incubated with poly- and monoclonal antibodies against C1q, C3c, TCC, DAF, MCP and CD59 diluted in PBS overnight at 4°C. Peroxidase-labelled rabbit anti-mouse IgG or swine anti-rabbit Ig diluted in PBS were subsequently applied for 60 min. The cytospin slides were finally treated with a diaminobenzidine (Sigma)-substrate-containing buffer. Control preparations were incubated with corresponding amounts of irrelevant mouse monoclonal antibodies or normal rabbit serum. All cytospin slides were counterstained with haematoxylin, mounted in Mowiol, and analysed using a Nikon light microscope (Melville, NY, USA).

### IgG responses in AF against ovarian cancer cell antigens

Ovarian cancer cell (Caov-3, PA-1, SK-OV-3 and SW626) membranes were isolated and solubilised. The membrane proteins were separated by SDS–PAGE, and immunoblotting was employed to examine if the AF samples contained IgG with specificity directed against ovarian cancer cell antigens. HUV-EC-C cells were used for comparison. Suspensions of the cells were obtained by incubating the cell cultures with 0.02% EDTA. After washing twice, the cells were incubated in 1 ml of ice-cold hypotonic lysis buffer (10 mM Tris and 10 mM NaCl, pH 7.4) containing protease inhibitors (C*ø*mplete™ Mini, Roche, Mannheim, Germany) for 10 min. The plasma membranes in suspension were disrupted with a teflon pestle in a glass homogeniser and further incubated for 10 min on ice. After removing the nuclei by centrifugation (twice at 1500 *g*, 5 min), the cell membranes were pelleted at 20 000 *g* for 15 min. The membrane pellets were run in SDS–PAGE slab gels (5–15%) under nonreducing conditions and transferred onto nitrocellulose sheets. The strips of the nitrocellulose sheets were incubated with AF samples diluted in PBS (1:800), and the bound IgG was detected with peroxidase-conjugated anti-human IgG and a chemiluminescence detection system.

## RESULTS

### Immunoglobulins, C components and regulators in AF

All the patients with neoplasms were previously untreated, and all underwent surgery. The clinical characteristics of the patients are summarised in Table 1.

To analyse factors needed in humoral immune responses, we initially measured the concentrations of IgG, IgM and C components in the AF samples (Table 2). The mean concentrations of IgG (8.66±2.58 g l^−1^) and IgM (0.35±0.12 g l^−1^) in the ovarian cancer patients’ AFs were lower than the average concentrations in normal human sera, despite the fact that the total protein concentrations in the malignant AFs were 93–140% of the serum values. In comparison, the concentrations of IgG in a patient with alcoholic liver cirrhosis (2.13 g l^−1^) and in a patient with chronic active hepatitis (2.74 g l^−1^) were approximately 1/4 of the lower limit measured for NHS (8.0–18.0 g l^−1^), while the IgM levels in these samples were within the same range as in the malignant AF.

The levels of C3 (0.79±0.25 g l^−1^) and C4 (0.15±0.05 g l^−1^) in the AF samples were approximately the same or at the lower limit measured for serum samples (0.5–1.5 and 0.15–0.50 g l^−1^, respectively). The concentrations of C3 and C4 in the nonmalignant AFs were below the threshold levels of the assays used for the measurements. The C1INH-level (0.21±0.04 g l^−1^) in malignant AFs was similar to the one present in normal serum (range: 0.21–0.24 g l^−1^). In contrast to most other parameters, the average combined level of factor H and FHL-1 (0.758±0.312 g l^−1^) in the AF from ovarian cancer patients was higher than that in NHS (0.461±0.063 g l^−1^) (Junnikkala *et al*, 2002). In comparison, the factor H/FHL-1 levels in the liver failure AF samples were very low (0.016 and 0.050 g l^−1^).

### Soluble forms of membrane bound C regulators in AF

Interestingly, in addition to C1INH and factor H/FHL-1, we detected soluble forms of C membrane regulators in AF. By immunoblotting, soluble MCP was detected in AF in seven out of 12 cases with ovarian cancer. The anti-MCP antibody reactive bands (58 and 68 kDa) comigrated with MCP from peripheral blood cells. No MCP was detected in AF from patients suffering from conditions other than ovarian cancer (Table 2).

Unlike MCP, no soluble form of DAF could be detected in any of the samples tested (Table 2), while erythrocyte extracts run in the same setups showed positive staining for DAF at 70 kDa.

**Figure 1:**
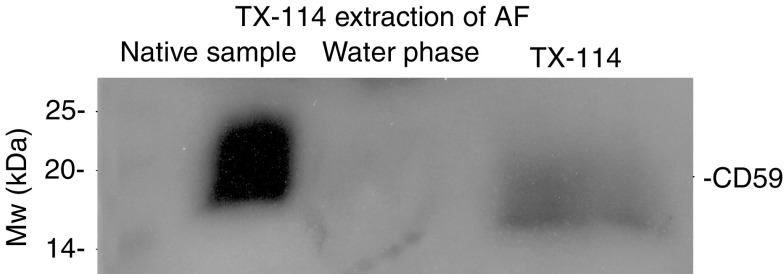
CD59 in ascites fluid (AF) has acyl-chains attached to it. The Triton X-114 (TX-114) partitioning was used to separate soluble and phospholipid-containing forms of CD59 in AF. TX-114 (0.5%) was added to cell-free AF at +4°C. After an incubation at +37°C, the detergent and water phases were separated by centrifugation. The native AF sample, detergent and water phases of AF were run in nonreduced SDS–PAGE (10%), transferred onto nitrocellulose membranes and probed with an anti-CD59 mAb (BRIC229). CD59 was detected principally in the detergent phase, indicating that the majority of CD59 molecules still contained membrane phospholipids.

A characteristic anti-CD59 reactive smear around 20 kDa was detected in all AF samples tested. A semiquantitative comparison of the amount of CD59 in the AF samples is shown in Table 2. To determine whether CD59 was linked to membrane phospholipids, AF samples from ovarian cancer patients were subjected to partitioning with the TX-114 detergent. As shown in Figure 1, CD59 was detected principally in the detergent phase that contains the membrane-associated form of CD59 (Lehto and Meri, 1993). The approximate relative amounts of CD59 in the aqueous and detergent phases were found to be 20 and 80%, respectively, as judged from the intensities of staining (*n*=7). Similar results were obtained when AF samples from the three patients with other gynaecological diseases were examined.

### Release of membrane C regulators from cultured ovarian tumour cells

**Figure 2:**
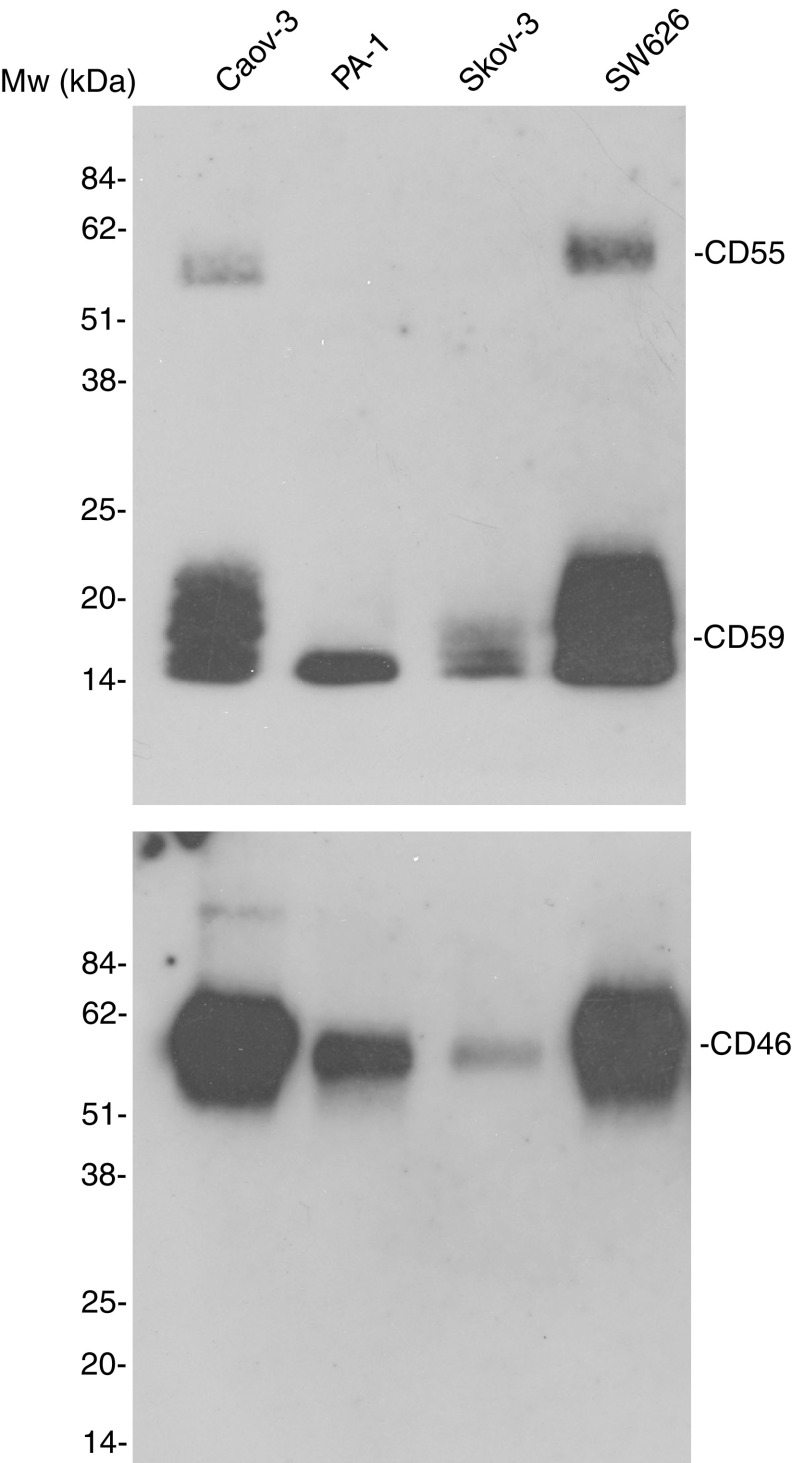
Immunoblotting analysis of C regulators in the concentrated cell culture supernatants of Caov-3, PA-1, SK-OV-3 and SW626 ovarian cancer cells. Confluent cultures of ovarian cancer cells were incubated with serum-free cell culture medium for 48 h. The centrifuged and filtered (0.45 *μ*m) cell culture supernatants were concentrated (100 ×). Aliquots (20 *μ*l) were electrophoresed in 10% nonreducing SDS–PAGE slab gels and blotted onto nitrocellulose membranes. The membranes were incubated with antibodies to CD46 (J4-48), CD55 (BRIC 230 or 216), CD59 (BRIC 229) and SP40,40.

To analyse whether soluble forms of C membrane regulators could originate from tumour cells, we examined concentrated cell culture supernatants from Caov-3, SK-OV-3, PA-1 and SW626 cells by immunoblotting (Figure 2). Our earlier FACS analysis showed expression of MCP and CD59 on all the four cell lines and DAF expression on the Caov-3, SK-OV-3 and SW626 cell lines, but not on the PA-1 cell line (Bjørge L *et al*, 1997a). Supernatants from Caov-3, PA-1 and SW626 cells showed two bands of 46 and/or 70 kDa that reacted with the anti-CD46 mAb J4-48. No such reactivity was seen in the concentrated SK-OV-3 medium. A 70 kDa band was detected when the SW626 supernatant was stained with the anti-CD55 mAbs BRIC216 and BRIC230. None of the supernatants from the other cell lines showed detectable CD55. The anti-CD59 mAb BRIC229 revealed bands of 21 kDa in the supernatants from all the cell lines. No bands could be detected in the negative control, where the primary antibodies were omitted.

### Haemolytic C activity and C activation products in the patients’ AF samples

The median haemolytic activity of the classical C pathway in AF (75 U ml^−1^) was within the range detected in NHS (50–100 U ml^−1^) (Table 3). In only thee samples, no classical pathway activity was detected (nos. 1, 14 and 16). Alternative pathway haemolytic activity in AF samples (95%) was also similar to values in serum (66–129%) (Table 3). No haemolytic activity of either pathway was detected in the non-neoplastic AFs.

To analyse whether C activation had occurred in the AF samples, the levels of C activation products C3a and SC5b-9 were measured. The data are shown in Table 3. The levels of C3a were clearly elevated (2552±1292 ng ml^−1^) in the ovarian cancer patient AF samples, as compared to values present in normal sera (26–146 ng ml^−1^) or in nonmalignant AF samples (240 and 1200 ng ml^−1^). The same was also the case for levels of SC5b-9 in AF samples. The ELISA measurements showed a mean SC5b-9 concentration of 6772 ng ml^−1^ in the ovarian tumour AF samples. However, the range was wide (0–20211 ng ml^−1^; Table 3). No SC5b-9 was detected in samples 7 and 11. In samples from patients with liver failure (nos. 17 and 18), the values were 2493 and 1113 ng ml^−1^, respectively. In conclusion, C activation to the terminal stage had occurred in most, but not all AF samples from ovarian tumour patients.

### Sensitivity of ovarian tumour cells to ascites-mediated cytotoxicity

**Figure 3:**
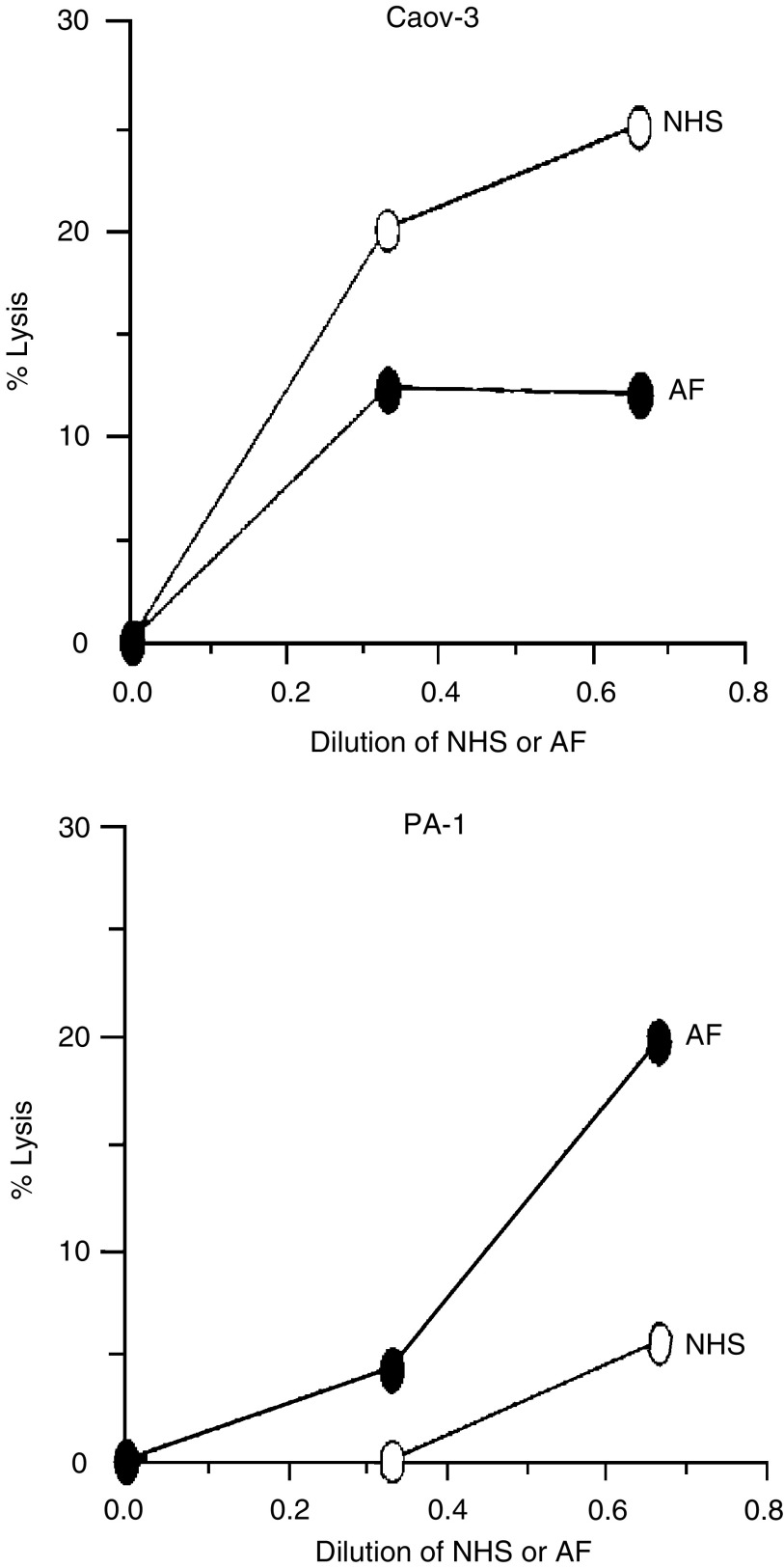
Complement-mediated lysis of PA-1 and Caov-3 cells with ascitic fluids from ovarian cancer patients. Caov-3 and PA-1 cells were labelled with ^51^Cr. After sensitising with the anti-CD59 C-activating mAb (YTH53.1; 350 *μ*g ml^−1^), the cells were exposed to ascites fluid (•) or normal human serum (○). The AF and serum dilutions are indicated on the *x*-axis. The released radioactivity was counted from the supernatants after a 30 min incubation at 37°C. Cell lysis was determined as a percentage of the maximum releasable radioactivity: ((released radioactivity−spontaneous release)/(maximum released radioactivity−spontaneous release)) × 100%.

The cytotoxic C activity in AF was examined by first sensitising ^51^Cr-labelled Caov-3 and PA-1 cells with a C-activating and CD59-neutralizing mAb (YTH53.1) and exposing the cells to AF. Normal human serum was used as a control source of C in the cell-killing assay. Maximal killing of Caov-3 and PA-1 cells was obtained by treating them with 1% NP-40 detergent. Both Caov-3 and PA-1 cells showed a weak dose-dependent lysis with NHS and AF in the assay. Typical examples are shown in Figure 3. During a 60 min incubation at +37°C, using saturating concentrations of YTH53.1, approximately 5 and 20% of the PA-1 cells were killed in the presence of 33 and 66% of AF (sample no. 20), respectively (Figure 3). In comparison, when PA-1 cells were subjected to C-mediated killing with NHS as a source of C, 2 and 6% of them became lysed in the presence of 33 and 66% of NHS, respectively. However, when the Caov-3 cell line was examined in the same assay, the tested serum sample showed stronger cytolytic activity than the AF sample (no. 15) (Figure 3). No lysis of Caov-3 and PA-1 cells was detected if YTH53.1 was omitted.

### Immunostaining of the ovarian tumour cells for membrane bound C regulators and C deposits

**Figure 4:**
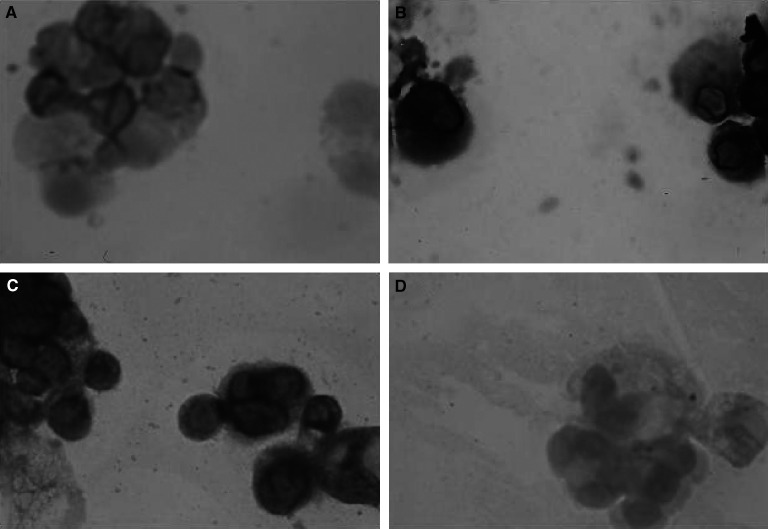
Immunohistochemical analysis of the expression of membrane bound C regulators on ovarian tumour cells isolated from AF. Cytotospin specimens were fixed and stained with the mAbs BRIC 216 (**A**), J4-48 (**B**) and BRIC 229 (**C**) directed against DAF, MCP and CD59, respectively. An irrelevant mouse IgG was used as a negative control (**D**). The bound mAbs were detected using an immunoperoxidase staining kit. Original magnification, × 200. Membrane cofactor protein and CD59 were strongly expressed by all the isolated tumour cells (**B** and **C**), while DAF expression was weaker and more heterogeneous (**A**).

**Figure 5:**
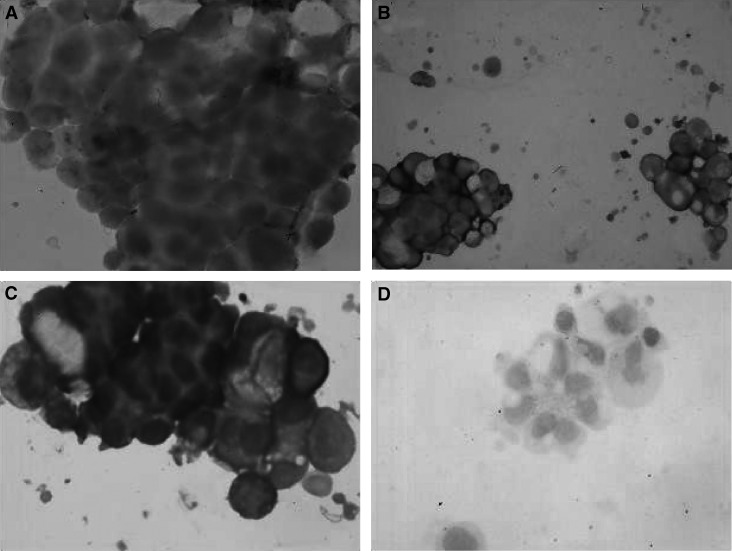
Immunohistochemical analysis of the C deposits on ovarian tumour cells isolated from AF. The cell specimens were stained with polyclonal antibodies against C1q (**A**) and C3d (**B**) and a mAb directed against a TCC neoantigen (**C**). Normal rabbit serum was used as a negative control (**D**). The bound Abs were detected using an immunoperoxidase staining kit, and the slides were counterstained with haematoxylin. Original magnifications, × 40 (**A**) and × 200 (**B**, **C** and **D**). Deposits of C1q (**A**) and C3d (**B**), but not of C5b-9 (**C**), could be detected. No reactivity was seen when the primary antibody was replaced with normal rabbit serum (**D**).

Isolated tumour cells from two patients were examined for C regulators and possible deposits of C components. Decay-accelerating factor was expressed on cells from both patients, although only 57% (sample no. 2) and 78% (sample no 10) of the tumour cells stained positive for DAF (Figure 4A). All the cells from both patients expressed both MCP and CD59 (Figure 4B and C). Although the intensity of staining was stronger for MCP than for CD59, the staining patterns were similar; both membranous and intracytoplasmic granular staining were seen. In comparison, DAF expression was clearly weaker than that of MCP and CD59. Deposits of C1q and C3d, but not of C5b-9, could be detected on isolated tumour cells from both samples (Figure 5). The staining pattern for C1q was a prominent circumferential, linear membrane staining, while the C3d staining was weaker and less distinct. No reactivity was seen when the primary antibody was replaced with control antibodies.

### Specific IgG responses in patients' AF against ovarian cancer cell antigens

**Figure 6:**
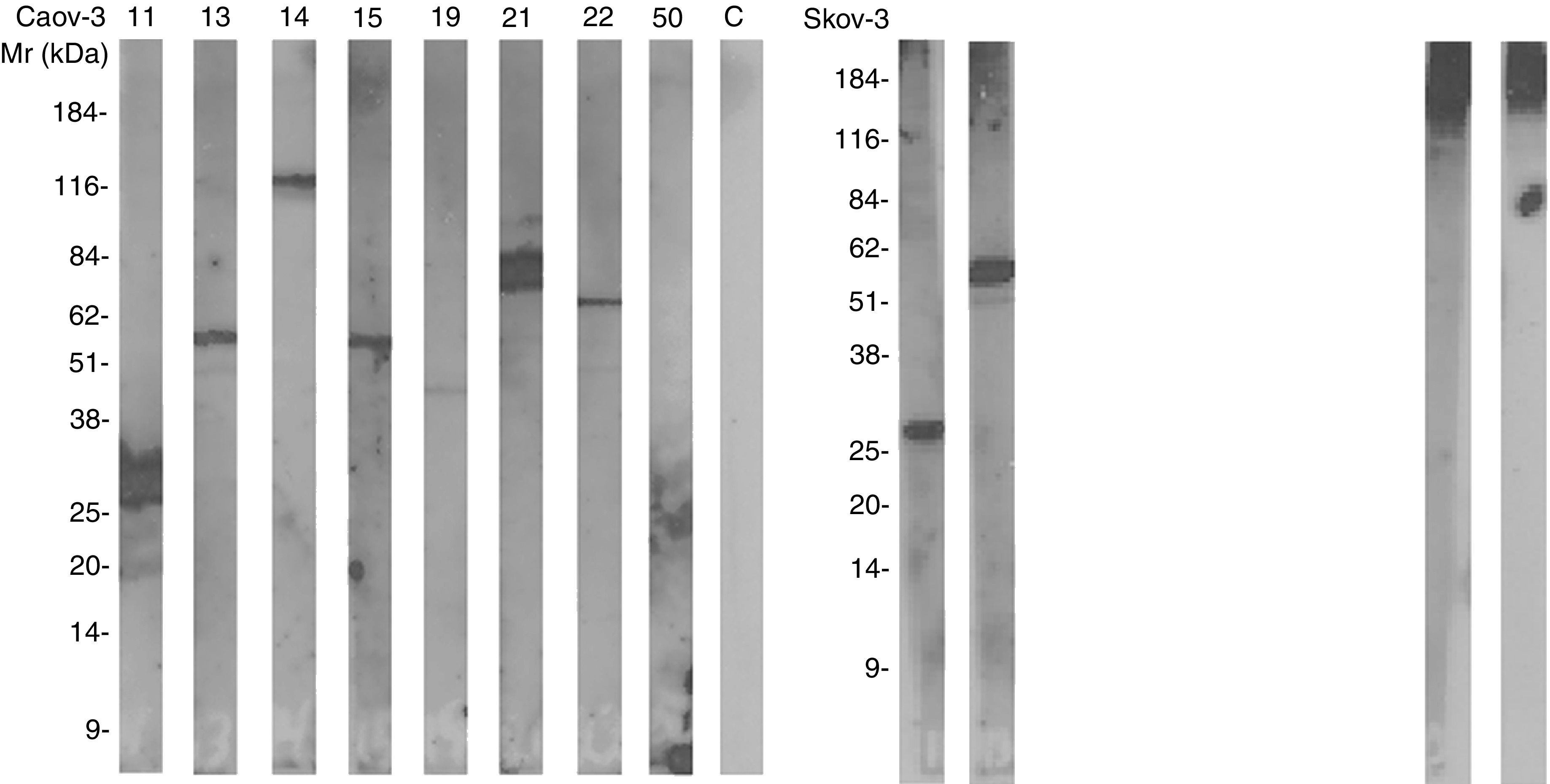
Immunoblotting analysis of IgG in AF reacting with ovarian cancer cell membrane antigens. Cell membranes from Caov-3, PA-1, SK-OV-3 and SW626 cells were isolated and electrophoresed in 5–15% SDS–PAGE gels and transferred onto nitrocellulose filters. The nitrocellulose strips were incubated with the AF samples (diluted 1 : 800). Bound IgG was detected by the enhanced chemiluminescence system.

In principle, C activation in AF against ovarian cancer cells could have become initiated by antitumour antibodies in the AF. Therefore, we studied whether the cancer patients’ AF contained IgG that would bind to ovarian tumour cell antigens. The membranes of Caov-3, PA-1, SK-OV-3 and SW626 cells were isolated, electrophoresed in 5–15% SDS–PAGE slab gels, blotted onto nitrocellulose filters and probed with dilutions of the AF samples. Immunoglobulins in the AF samples from the ovarian cancer patients reacted with several cell membrane antigens. IgG in the AF sample no. 11 reacted with a 25 kDa antigen in Caov-3, PA-1 and SK-OV-3 cell membranes. The AF sample no. 13 reacted with a 55 kDa antigen in Caov-3, PA-1 and SK-OV-3 cell membranes. The IgG in the AF sample no. 21 reacted with a 70 kDa antigen in Caov-3, PA-1 and SK-OV-3 cell membranes. In addition, AF IgG from six patients (nos. 13, 14, 19, 20, 23 and 24) detected a 48 kDa antigen in PA-1 cells (Figure 6). In contrast, IgG in the AFs from the control patients with liver failure did not detect any antigens from the Caov-3, PA-1 or SK-OV-3 cell membranes.

## DISCUSSION

In this study, we have observed tumour-reactive antibodies of the IgG class, and a functionally active C system in the AF of most of the examined patients with ovarian cancer. High levels of C activation products in the AF samples and deposits of C1q and C3 on the isolated tumour cells indicate that the AF C system has become activated *in vivo*. The tumour cells expressed membrane bound C regulators on their surfaces and secreted their soluble forms, as well as other soluble C inhibitors into their local environment. These inhibitors are likely to restrict the cytotoxic activity of AF against tumour cells. Our results suggest that although the ovarian tumour cells in the malignant AF fluids are surrounded by a functional C system, they are protected against C activation. The tumour cells have C inhibitors on their cell membranes and may also create a microenvironment where fluid phase C activation is regulated. Therefore, if the ovarian tumour cells are to be killed by tumour-specific mAbs and AF C, their intrinsic C resistance, at least in part, must be overcome. This could be achieved, in principle, with specific C regulator neutralising antibodies.

The protein concentrations in malignant ovarian cancer AF samples were 94–140% of those in serum, while the protein levels in nonmalignant AFs were only 11–13.5% of serum values. These data are similar to those reported earlier by others, and illustrate the exudate and transudate natures of malignant and nonmalignant AFs, respectively (Parsons *et al*, 1996). The substantially high protein content in malignant AF is likely due to an increased permeability of small blood vessels, release and shedding of proteins from tumour cells, inflammatory and mesothelial cells (Parsons *et al*, 1996), while the nonmalignant AF is a pure transudate.

The antibodies of the IgG and IgM classes were present in malignant AF at median concentrations of 8.12 and 0.33 g l^−1^, respectively. IgM and IgG in AF might be derived both from serum, and/or synthesised locally as a result of a local immune response. The study by Guzman *et al* (1988) suggests that a portion of ascitic Igs could be of local origin as AF contains both B cells and plasma cells (Nakamura *et al*, 1978). As shown in Figure 5, some of the AF IgG bound to distinct surface structures on the ovarian cancer cells. The responses seemed to be individual as no specific uniform binding pattern profile could be obtained for the patient group as a whole. Antibodies present in the AF could act as activators of the C system.

A functionally active C system was detected in the AF of patients with ovarian cancer. In line with earlier studies (Runyon *et al*, 1985; Mal *et al*, 1991), the amounts of C3 and C4 were comparable to those present in NHS. Both factors can be produced locally by inflammatory and epithelial cells. To our knowledge this is the first study examining the cytotoxic activity of C in malignant AF. Our results show that a functionally active C system is present in the AF. Classical and alternative pathway haemolytic activities were seen in nine out of 12 and 12 out of 12 of the malignant AFs, respectively. For most positive samples, the activity was at the level of that present in NHS. In comparison, no such activity could be found in the nonmalignant AF samples.

The presence of soluble C3a and SC5b-9 showed that the C system in AF had become activated *in vivo* in many of the patients. The concentrations of C3a and SC5b-9 were much higher in patients with a neoplastic disease than in patients with liver failure. Furthermore, on isolated tumour cells deposits of C1q and C3d, but not of C5b-9, could be detected. This is an original observation, and one of the first reports to show C deposition on tumour cells *in vivo*. Complement activation may be due to classical pathway activation initiated directly by membrane-bound antibodies or indirectly by immune complexes in the AF. Immune complex-induced C activation may lead to a bystander attack against adjacent cells. Alternatively, activation may have occurred by antibody-independent mechanisms, either via the alternative pathway, or via a direct interaction of C1q with exposed cell constituents like perturbed cellular or mitochondrial membranes or chromatin. Tumour cells that have become detached from their original sites may undergo apoptotic (or necrotic) changes and expose binding sites for C1q. This together with a reduced ability to control C could lead to C activation.

To examine whether C in AF could kill viable tumour cells, we tested the cytotoxic activity of the AF samples in a targeted C-mediated killing assay. Different combinations of ovarian tumour cell lines and AF samples were tested. By using a specific C-activating mAb against the cell surface CD59-antigen (YTH53.1; IgG2b), we found that it was possible to use the AF as a cytotoxic C source. For most of the AF samples examined, the cytotoxic activities were weaker than in the NHS samples tested for comparison.

To investigate whether the tumour cells' relative resistance to C could be due to the expression of membrane regulators CD46, CD55 and CD59, we performed immunohistochemical analysis of tumour cells isolated from two patients with ovarian cancer. As shown in Figure 4, MCP and CD59 were strongly expressed on all the tumour cells. Decay-accelerating factor was more heterogeneously expressed as only 58 and 75% of the tumour cells showed positive staining. The staining intensities varied both between the samples and between the tumour cells from the individual patient. The latter heterogeneity could, in part, be due to varying stages in the tumour cells' life cycles or to genomic instability that is a general property of neoplastic cells (Fleuren *et al*, 1995). The expression patterns found on the isolated and purified cancer cells from the AF were similar to the expression patterns found earlier in solid ovarian tumours (Bjørge L *et al*, 1997a).

We have previously shown that the amounts of alternative pathway inhibitors FH/FHL-1 are higher in AF samples than in sera (Junnikkala *et al*, 2002). The concentrations of FH/FHL-1 in AF were relatively high (Table 2); nearly twice of those in sera. We have observed that it is specifically the concentration of FHL-1 that is high in AF, and that functionally active FH and FHL-1 molecules are synthesised by the ovarian tumour cells (Junnikkala *et al*, 2002). The small FHL-1 molecule may thus act as a ‘quick-response’ protective molecule to suppress C activation. Released soluble MCP may also have a similar, although a weaker activity in the AF.

The amount of cell-free CD59 was found to be higher in AF than in sera and in AF from patients with liver cirrhosis (Table 2). We have shown earlier that ovarian and breast tumours of various histological types all express high levels of CD59 (Hakulinen and Meri, 1994; Bjørge L *et al*, 1997a;). The AF samples were obtained from patients with a big tumour burden. The observation that AF from patients with other large-sized gynaecological tumours of different histological types (both malignant and benign) also contained high levels of CD59 suggests that the release of CD59 from cells is related to tumour mass rather than to the malignancy itself.

The full inhibitory activity of CD59 requires the presence of an intact phospholipid tail that can incorporate into lipid bilayers. Using a Triton X-114 phase separation method, an average of 80% of AF CD59 was found to represent a form with anchor-associated phospholipid. Similarly, phospholipid-tailed CD59 has been found principally in amniotic fluid (Rooney and Morgan, 1992), seminal plasma (Rooney *et al*, 1993) and in milk (Hakulinen and Meri, 1995). A proportion of CD59 may also be shed in small membrane vesicles. The presence of a high level of phospholipid-containing CD59 may in part explain the results of the functional analysis showing that AF in general has a weaker C activity than NHS.

The presence of soluble forms of C membrane regulators in AF is assumed to be due to release from tumour cells. The immunoblotting experiments showed that the cultured ovarian cancer cells can shed the C regulators CD46, CD55 and CD59 into the culture medium. There is evidence that this is also the case with tumours derived from other tissues (Bjørge L *et al*, 1994, 1996; Jurianz *et al*, 1999; Hakulinen *et al*, 2004). It is likely that the C regulators in AF, at least in part, are derived from the tumours themselves. The secretion of both soluble C regulators, like FH/FHL-1, and the release of membrane regulators can thus provide additional resistance to ovarian tumours in the AF or at least restrict the activity of the C system within the AF.

The presence of C activators and a functional C system in malignant AF suggests that C attack against tumour cells represents a partially operative immunological surveillance mechanism. We have, however, shown that the AF C system has an insufficient capacity to mediate effective C-mediated tumour cell killing. The expression of membrane bound C regulators on tumour cells and the release of C regulators may at least partly explain the resistance. In addition, aggregated malignant cells are more difficult to kill by C than single cells (Bjørge L *et al*, 1997b; Hakulinen and Meri, 1998), further contributing to the tumour escape.

Recently, Ng *et al* (2002) have reported that the intraperitoneal injection of the C activating mAb rituximab is an effective measure to control recurrent abdominal ascites in non-Hodgkin's lymphoma. In the case of ovarian cancer, the presence of tumour cells within the peritoneal cavity surrounded by a functional C system also makes this tumour type an attractive target for local monoclonal antibody therapy (Markman, 1998). Although clinical trials so far have failed to lead to any consistent response pattern, it is possible that such therapy could be of considerable benefit. Soluble as well as membrane regulators of C restrict tumour cell lysis. However, the tumor cell killing by mAbs could be improved if the activity of the C system within the peritoneal space can be enhanced and the sensitivity of the tumour cells increased, for example, by temporarily blocking C inhibitors. As shown in the present study, the AF with its active C system could provide effector mechanisms for mAb-mediated therapy of ovarian tumours.

## Figures and Tables

**Table 1:** Clinical characteristics of the patient group

**Patient no.**	**Age**	**Diagnosis**	**Stage**	**Histology**
1	71	Ovarian cancer	IIIb	Serous cystadenocarcinoma
2	60	Ovarian cancer	IIIc	Serous cystadenocarcinoma
3	75	Ovarian cancer	III	Serous cystadenocarcinoma
4	30	Ovarian cancer	IIIc	Endometroid adenocarcinoma
5	44	Ovarian cancer	IV	Papillary serous cystadenocarcinoma
6	52	Ovarian cancer	Ib	Endometroid adenocarcinom
7	67	Ovarian cancer	IIIc	Papillary serous cystadenocarcinoma
8	59	Ovarian cancer	IIIc	Papillary serous cystadenocarcinoma
9	72	Ovarian cancer	IV	Papillary serous cystadenocarcinoma
10	72	Ovarian cancer	IV	Papillary serous cystadenocarcinoma
11	74	Ovarian cancer	IIIc	Papillary serous cystadenocarcinoma
12	57	Ovarian cancer	IIIc	Mucinous adenocarcinoma
				
13	65	Endometrial cancer	Ic	Endometroid adenocarcinoma
14	56	Fallopian tube cancer	IIIc	Adenocarcinoma from the left tube
15	50	Breast cancer		Serous cystadenocarcinoma
16	59	Cystadenoma in the left ovary		Serous cystadenoma
17	68	Alcoholic liver cirrhosis		
18	64	Chronic active hepatitis (seronegative), liver cirrhosis and hepatorenal syndrome		

**Table 2:** Levels of immunoglobulins, total protein, complement factors and regulators in ascites fluids

**Diagnosis**	**No.**	**IgG (g l^−1^)**	**IgM (g l^−1^)**	**Protein (g l^−1^)**	**C3 (g l^−1^)**	**C4 (g l^−1^)**	**C1INH (g l^−1^)**	**FH/FHL-1 (g l^−1^)**	**MCP**	**DAF**	**CD59**
Ovarian cancer	1	7.698	0.329	84.0	0.466	<0.064	0.127	0.618	+ (U)^a^	−	+++
	2	5.431	0.371	64.0	0.777	0.205	0.253	0.677	+	−	+
	3	7.356	0.225	79.5	0.673	0.200	0.159	0.718	+	−	+++
	4	5.106	0.211	74.5	0.786	0.136	0.240	1.498	+ (L)	−	+
	5	8.409	0.377	91.5	1.388	0.205	0.213	0.473	−	−	+
	6	12.86	0.311	96.5	0.436	0.113	0.201	1.148	−	−	++
	7	7.293	0.315	86.5	0.929	0.190	0.241	0.543	−	−	++
	8	10.00	0.377	64.5	0.900	0.160	0.262	0.767	−	−	+
	9	8.929	0.338	82.5	0.687	0.123	0.198	0.871	+ (U)	−	++
	10	13.66	0.659	77.0	0.620	0.102	0.184	1.169	+ (U)	−	+
	11	7.825	0.445	91.5	0.898	0.125	0.173	0.375	+ (U)	−	+
	12	9.329	0.267	89.5	0.961	0.194	0.215	0.941	−	−	++
	Mean±s.d.	8.66±2.58	0.35±0.12	81.79±10.40	0.79±0.25	0.15±0.05	0.21±0.04	0.758±0.312			
											
Other gynaecological neoplasms	13	6.818	0.311	64.5	0.903	0.130	0.215	0.700	−	−	++
	14	38.75	0.516	107.5	0.889	0.104	0.175	0.821	−	−	++++
	15	<0.042	0.710	41.0	0.777	0.131	0.168	0.395	−	−	+
	16	8.071	0.346	84.5	0.772	0.200	0.144	0.424	−	−	+++++
Liver failure	17	2.13	0.250	9.3	<0.177	<0.064	<0.031	0.016	−	−	−
	18	2.74	0.416	7.9	<0.177	<0.064	<0.031	0.050	−	−	−
											
Serum	NHS	8.0–18.0	0.6–2.7	62–76	0.5–1.5	0.15–0.50	0.12–0.24	0.335–0.587	−	−	−

^a^U=upper Mw form predominating; L=lower Mw form predominating.

For methods, see Materials and Methods. The values in normal human serum samples are shown for comparison. They represent mean±2 s.d. in 100 healthy blood donors.

**Table 3:** Haemolytic C activities and C activation product levels in AF samples

**Diagnosis**	**Sample no.**	**CH100CL (U ml^−1^)**	**CH100AP (% normal)**	**C3a (ng ml^−1^)**	**SC5b-9 (ng ml^−1^)**
Ovarian cancer	1	<25	35	5100	2546
	2	40	95	2750	11996
	3	60	115	1800	20211
	4	100	80	3600	18928
	5	100	100	3600	12414
	6	<25	95	1500	1511
	7	<25	80	1100	<10
	8	100	100	2000	2575
	9	140	100	3000	4049
	10	90	70	1375	2955
	11	70	40	1000	<10
	12	80	120	3800	4067
	Mean±s.d.	68.13±41.53	85.83±26.61	2552±1292	6771±7208
					
Other gynaecological neoplasms	13	70	65	2750	<10
	14	<25	50	6300	2946
	15	90	60	7300	12659
	16	<25	65	3500	12307
Liver failure	17	<25	—	240	2493
	18	<25	—	1200	1133
					
Serum/range		50–100	66–129		
					
Plasma/range				26–146	<100
